# Penile Entrapment Caused by Five Metallic Rings: A Case Report

**DOI:** 10.7759/cureus.88995

**Published:** 2025-07-29

**Authors:** Hector R Gonzalez-Carranza

**Affiliations:** 1 Urology, Hospital Angeles Metropolitano, Mexico City, MEX

**Keywords:** ischemia of the penis, paraphilic disorders, penile injury, penile strangulation, penile trauma, penis, sexual intercourse, sexual paraphilia, sexual risk behaviors, vigorous sexual activity

## Abstract

Penile strangulation is a rare urological emergency that can lead to severe complications such as ischemia, necrosis, and urethral injury. We present a case of a 76-year-old man who presented to the emergency department with severe penile pain and swelling after applying five metallic rings to enhance erection during sexual intercourse. The patient had a history of erectile dysfunction and had been using phosphodiesterase inhibitors with a moderate response. Physical examination revealed significant edema distal to the glans, but capillary refill and sensation were intact, indicating preserved perfusion. Initial attempts to remove the rings with lubrication and manual manipulation were unsuccessful due to the extent of the swelling. A ring cutter was used to cut through each ring while protecting the underlying tissue, and the entire process lasted approximately 90 minutes. After removal, no signs of laceration, urethral injury, or necrosis were observed. The patient was discharged with anti-inflammatory medication and oral antibiotics. This case highlights the challenges associated with the management of penile strangulation injuries, particularly when multiple metallic objects are involved. Prompt evaluation, creative procedural approaches, and multidisciplinary collaboration are critical for minimizing morbidity. Elderly individuals engaging in sexual experimentation should be informed about the potential risks associated with the use of constricting devices.

## Introduction

Penile strangulation is a urological emergency that can lead to ischemia, necrosis, or urethral injury [1, 2]. First described by Gauthier in 1755, this condition has been reported all over the world, with a variety of causative constricting objects, such as rings, bottles, and metallic tubes, used to enhance sexual activity [3-5].

Although more common in younger or middle-aged men, elderly patients also present with such emergencies. Recent studies highlight a wider use of phosphodiesterase type 5 inhibitors and greater openness about sexuality in older age. This has increased the prevalence of sexual activity among individuals over 70 years of age. According to the National Social Life, Health, and Aging Project (NSHAP), approximately 54% of men aged between 75 and 85 remain sexually active [6]. Devices such as the one presented in this case may be used by individuals seeking novel sexual experiences, addressing erectile dysfunction, psychiatric illness, cognitive decline, or paraphilic tendencies [7-10].

The removal of constrictive objects can be technically challenging, especially when the foreign object is metallic, rigid, or thick. Successful intervention requires specialized cutting tools and interdisciplinary approaches involving urology and emergency medicine [11, 12].

This report details the case of a 76-year-old man who presented to the emergency department with penile strangulation caused by five metallic rings. 

## Case presentation

**Figure 1:**
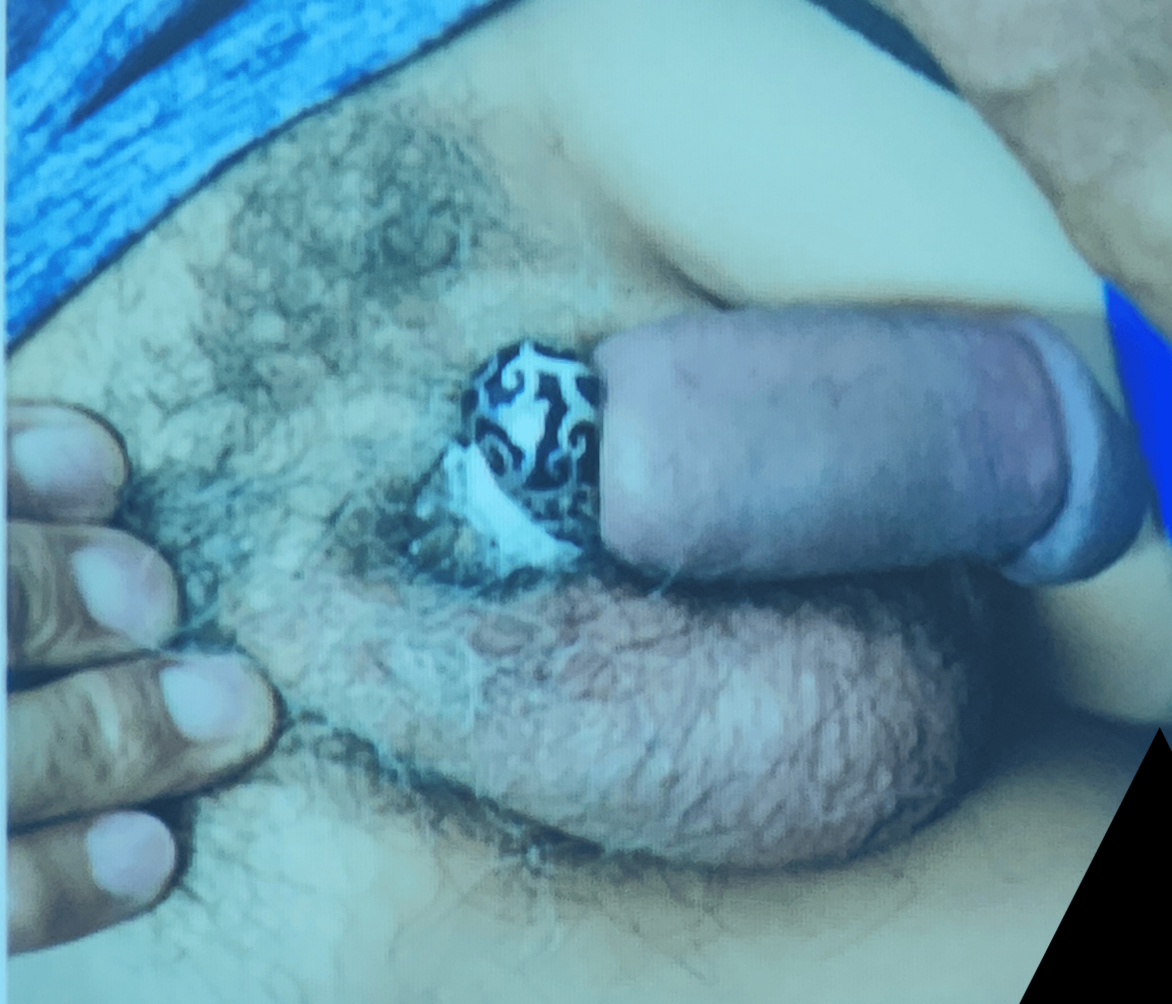
Penile swelling caused by entrapment in five metallic rings

A 76-year-old man presented to the emergency department with severe penile pain and swelling after applying five metallic rings to enhance erection during sexual intercourse. He had the rings on for approximately five hours prior to the presentation and was unable to remove them because of severe swelling and discomfort (Figure 1).

He had no history of relevant psychiatric conditions or any prior episodes of self-injurious behaviors, but he reported having erectile dysfunction for the past 11 years and had been under treatment with phosphodiesterase inhibitors with a moderate response.

On physical examination, the patient was alert and hemodynamically stable. Genital inspection revealed five metallic rings, approximately 1.5 cm in width each, encircling the base of the penis, distal to the glans, causing significant edema. Capillary refill was less than two seconds, and sensation was intact, suggesting preserved perfusion.

**Figure 2:**
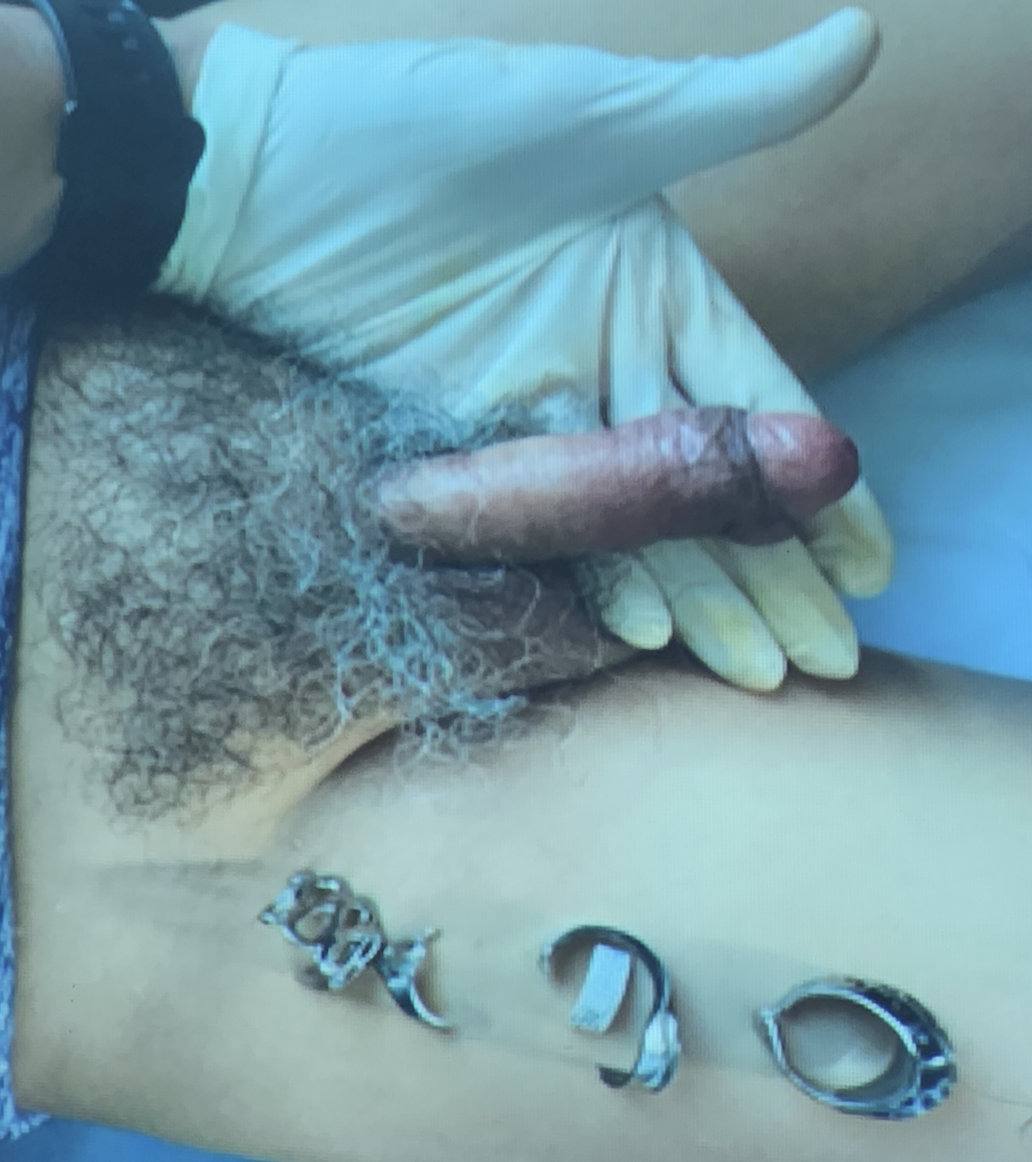
Penis after removal of constricting rings

Initial attempts to remove the rings with lubrication and manual manipulation were unsuccessful because of the extent of the swelling, necessitating the use of a manual ring cutter to cut through each ring while protecting the underlying tissue with gauze. This specific type of ring cutter was chosen for its enhanced control, which allows greater precision while cutting through the rings. Pain was managed with a single 30 mg sublingual dose of ketorolac. The entire process lasted approximately 90 minutes (Figure 2).

After removal, the penis was inspected for signs of injury. No signs of laceration, urethral injury, or necrosis were observed. The patient was discharged with anti-inflammatories and oral antibiotics. On follow-up, the patient reported no changes in erectile function or other associated symptoms.

## Discussion

Penile strangulation injuries are surgical emergencies that require prompt and often creative interventions. Although rare, this condition necessitates immediate urological management to avert potentially devastating outcomes such as ischemia, necrosis, or gangrene [13].

Metallic objects, particularly rigid and thick objects such as steel rings, present a unique challenge. The duration of entrapment and the inability of common tools to penetrate hard materials can significantly delay the intervention. In this case, the presence of five rings compounded the complexity, making it particularly rare (Table 1) [14].

**Table 1:** Device characteristics complicating extraction Source: [7]

Factor	Impact on management
Material hardness	Metallic > plastic > elastic
Thickness	Thicker objects require mechanical cutting
Number of objects	Multiple rings increase procedural time
Duration in place	Longer time leads to more swelling
Location	Proximal placement near base harder to access

Psychological or paraphilic underpinnings, though not explicitly present in this patient's history, are frequently associated with such incidents, as some individuals might use these devices in an attempt to prolong erections or for sexual gratification, sometimes leading to accidental entrapment (Table 2) [15, 16].

**Table 2:** Indications for psychiatric referral after penile constriction injury Source: [8, 9]

Clinical feature	Rationale
Recurrent penile injuries	Possible compulsive or paraphilic behavior
Self-harm or suicidal ideation	Underlying psychiatric illness
Insertion of multiple or dangerous objects	Risk-taking behavior
Known psychiatric diagnosis	May benefit from coordinated care

## Conclusions

This case highlights the rare but serious consequences of penile constriction injuries, particularly when multiple metallic objects are involved. Prompt evaluation, creative procedural approaches, and multidisciplinary collaboration are critical for minimizing morbidity. Elderly individuals engaging in sexual experimentation should be informed about the potential risks associated with the use of constricting devices.
